
JOURNAL INFORMATION
==============================
NLM Title Abbreviation: ScientificWorldJournal
Journal ID: ScientificWorldJournal
NLM Journal ID: 101131163
Journal ID: TSWJ

The Scientific World Journal

ISSN: 2356-6140
EISSN: 1537-744X
Publisher: Wiley

ARTICLE INFORMATION
==============================
PMCID: PMC6084514
DOI: 10.1100/tsw.2001.429
Article ID: 794832
Article version: 1
Subjects: Extended Abstract

Transgenic Studies on the CGRP-Receptor Component Protein (RCP)

Mnayer Laila O. 1
Rosenblatt Mark I. 1
Dickerson Ian M. 1 2
1 Department of Biochemistry, University of Miami School of Medicine, Miami, FL, USA
2 Department of Physiology and Biophysics, University of Miami School of Medicine, Miami, FL, USA
Publication date: 2001
Electronic publication date: 2001 Nov 21
Volume: 1
First page: 44
Last page: 44
Copyright: Copyright © 2001 Laila O. Mnayer et al.
Copyright year: 2001
License: This is an open access article distributed under the Creative Commons Attribution License, which permits unrestricted use, distribution, and reproduction in any medium, provided the original work is properly cited.
License URL: https://creativecommons.org/licenses/by/3.0/

Funding: National Institutes of Health DK52328

==============================
4th International Meeting on Calcitonin Gene-Related Peptide (CGRP)
*TheScientificWorld* (2001) 1(S1), 44
ISSN 1532-2246; DOI 10.1100/tsw.2001.429

### TRANSGENIC STUDIES ON THE CGRP-RECEPTOR COMPONENT PROTEIN (RCP)

Laila O. Mnayer[1], Mark I. Rosenblatt[1], and Ian M. Dickerson[1,2]
[1]Department of Biochemistry, University of Miami School of Medicine, Miami, FL, USA;
[2]Department of Physiology and Biophysics, University of Miami School of Medicine, Miami, FL, USA

The CGRP-receptor component protein (RCP) is a 17-kDa intracellular peripheral membrane protein that is required for signal transduction by the calcitonin receptor-like receptor (CRLR). We have demonstrated that RCP is expressed in tissues and cell lines containing CGRP receptors, and that RCP expression is regulated in parallel with the biological efficacy of CGRP *in vivo*. We have made stable cell lines which express an antisense RCP cDNA, and have shown that loss of RCP protein correlated with loss of CGRP signal transduction, but not CGRP binding. RCP thus represents a new class of protein required for CGRP-mediated signal transduction. We are generating RCP-deficient mice using homologous recombination to extend these cell culture studies into an *in vivo* model. These RCP-null mice will have important uses for development of therapeutic strategies targeted at migraine, hypertension, and chronic pain.

To generate RCP-deficient mice by homologous recombination we have constructed a targeting vector that replaces the coding region of the RCP gene with a cassette containing the enhanced green fluorescent protein (EGFP) and the positive selectable marker neomycin phosphotransferase (neo). This cassette is flanked by genomic sequences that we have cloned from mouse genomic DNA. The placement of the EGFP gene under the control of the RCP promoter in recombinant mice offers a unique opportunity to study the expression of RCP in different adult and embryonic tissues, in addition to studying the effects of deletion of RCP on CGRP physiology. This work was supported in part by NIH DK52328 (IMD).
